# Inverse Comorbidity between Down Syndrome and Solid Tumors: Insights from In Silico Analyses of Down Syndrome Critical Region Genes

**DOI:** 10.3390/genes14040800

**Published:** 2023-03-26

**Authors:** Kwadwo Fosu, Jude Tetteh Quarshie, Kwabena Amofa Nketia Sarpong, Anastasia Rosebud Aikins

**Affiliations:** 1 Department of Biochemistry Cell and Molecular Biology, College of Basic and Applied Sciences, University of Ghana, Legon, Accra P.O. Box LG 54, Ghana; 2 West African Centre for Cell Biology of Infectious Pathogens, Legon, Accra P.O. Box LG 54, Ghana

**Keywords:** down syndrome, breast cancer, lung cancer, ETS2, RCAN1

## Abstract

An inverse comorbidity has been observed between Down syndrome (DS) and solid tumors such as breast and lung cancers, and it is posited that the overexpression of genes within the Down Syndrome Critical Region (DSCR) of human chromosome 21 may account for this phenomenon. By analyzing publicly available DS mouse model transcriptomics data, we aimed to identify DSCR genes that may protect against human breast and lung cancers. Gene expression analyses with GEPIA2 and UALCAN showed that DSCR genes *ETS2* and *RCAN1* are significantly downregulated in breast and lung cancers, and their expression levels are higher in triple-negative compared to luminal and HER2-positive breast cancers. KM Plotter showed that low levels of *ETS2* and *RCAN1* are associated with poor survival outcomes in breast and lung cancers. Correlation analyses using OncoDB revealed that both genes are positively correlated in breast and lung cancers, suggesting that they are co-expressed and perhaps have complementary functions. Functional enrichment analyses using LinkedOmics also demonstrated that *ETS2* and *RCAN1* expression correlates with T-cell receptor signaling, regulation of immunological synapses, TGF-β signaling, EGFR signaling, IFN-γ signaling, TNF signaling, angiogenesis, and the p53 pathway. Altogether, *ETS2* and *RCAN1* may be essential for the development of breast and lung cancers. Experimental validation of their biological functions may further unravel their roles in DS and breast and lung cancers.

## 1. Introduction

Down syndrome (DS) is the most common chromosomal disorder associated with intellectual disability. It occurs in approximately 1 of 800 births worldwide, with an ever-increasing lifetime prevalence as the global population increases. In the United States, DS accounts for nearly 5000 live births annually, and more than 200,000 persons are living with the disorder [1,2]. In Europe, the live birth prevalence of DS from 2011 to 2015 was 10.1 per 10,000 live births [3]. The prevalence of DS is influenced by maternal age at conception, which varies between countries and ethnicities. This variation—coupled with disparities in childhood survival and poor record keeping especially in under-developed countries—negatively impacts the precise calculation of a global estimate [4].

Symptoms range from physical defective characteristics such as craniofacial abnormalities (i.e., small head and ears, flattened facial profile), short neck, short stature, larger-than-average tongue, poor muscular tone, and one rather than two wrinkles across the palm. Other symptoms include delayed social and emotional development, difficulties with abstract reasoning and problem-solving, short-term memory, focus and concentration problems, learning impairment, and delay in language and speech development [2,4].

DS is caused by trisomy of *Homo sapiens* chromosome 21 (HSA21), i.e., the presence of an extra copy of a complete or partial HSA21. A free trisomy 21 (meiotic nondisjunction leading to the presence of 47 chromosomes) is present in 95% of individuals with DS, translocation (occurs with one HSA21 attached to HSA14, HSA21, or HSA22) accounts for trisomy 21 in 3 to 5% of affected individuals, and mosaicism for trisomy 21 (an extra copy of HSA2 present in some, but not all of the body cells) occurs in ~2% of individuals with DS [1,2,4]. The HSA21 is the smallest human autosome: it is approximately 46.7 million bp long and comprises 738 genes, of which 233 are protein-coding genes, while 505 encode microRNAs, long-noncoding RNAs, immunoglobulin genes and other regulatory elements [5]. The protein-coding genes encode for cell adhesion molecules, cell cycle regulators, transcription factors, kinases, ion channels, proteins involved in nucleic acid processing and/or modification, etc. [6]. On the long arm of the HSA21 is a region known as the Down Syndrome Critical Region (DSCR). The DSCR has an approximate length of 5.4 Mb, is located between bands 21q22.13 and q22.2 of HSA21, and harbors approximately 33 genes that are hypothesized to be responsible for the symptoms that collectively cause the DS phenotype [7].

Advanced maternal age at conception is a major risk factor for DS; pregnancies in women over 35 years are more likely to be affected by DS than pregnancies in women at a younger age [1]. This risk is associated with nondisjunction of HSA21 during meiosis I, meiosis II, or postzygotic mitotic divisions of oocytes [8,9]. Additionally, specific altered recombination patterns such as single pericentromeric or telomeric exchanges in meiosis associated with maternal age contribute to these types of errors [10]. Environmental factors also influence the risk of nondisjunction but are difficult to identify due to the problem of defining the exposure, dosage, and timing of each factor [4]. Environmental factors that contribute to DS include tobacco use, folate metabolism, oral contraceptive use and maternal socioeconomic status [11]. 

Due to the chromosomal aberration which results in DS, it is thought to be a predisposing factor for certain health conditions such as anxiety disorders, autoimmune diseases, epilepsy, early-onset Alzheimer disease, hypothyroidism, obstructive sleep apnea, and recurrent infections [4,12]. Surprisingly, epidemiological data show that while persons with DS are more (10–30 times) likely to be diagnosed with hematological malignancies such as acute myeloid, acute lymphoblastic and acute megakaryoblastic leukemias, solid tumors are less likely to occur within the DS populace [13]. Researchers initially recognized this phenomenon in the 1940s when it was noticed that persons with DS appeared to have a lower risk of developing cancer. Since then, some investigations have supported this finding; notably, for lung cancer, prostate cancer, and breast cancer. In a study of persons with DS in Denmark, a very low risk of breast and lung cancers was determined [14]. Another study of DS individuals in Finland reported a low incidence of breast cancer [15]. 

This inverse comorbidity has been attributed to higher susceptibility to apoptosis in DS, which results in cell death instead of tumorigenesis following cell injury [14]. Another philosophy ascribes this phenomenon to aberrant histone modification observed in DS individuals. Although this hypothesis requires further investigation, it has been shown that aberrant methylation in DS persons leads to mitochondrial dysfunction which results in cell death, killing malignant cells [16,17]. Again, the inverse comorbidity has been attributed to the overexpression in specific DSCR genes due to the presence of the extra HSA21 and the downstream consequences of their overexpression [4]. For example, DS is characterized by the overexpression of antiangiogenic markers found on HSA21 which halt the progression of solid tumors that directly depend on angiogenesis. Such antiangiogenic markers include Col18A1 (collagen Type XVIII α 1 Chain), RCAN1 (regulator of calcineurin 1/calcipressin-1), and DYRK1A (dual-specificity tyrosine-phosphorylation regulated kinase 1A) [6]. 

**Figure 1:**
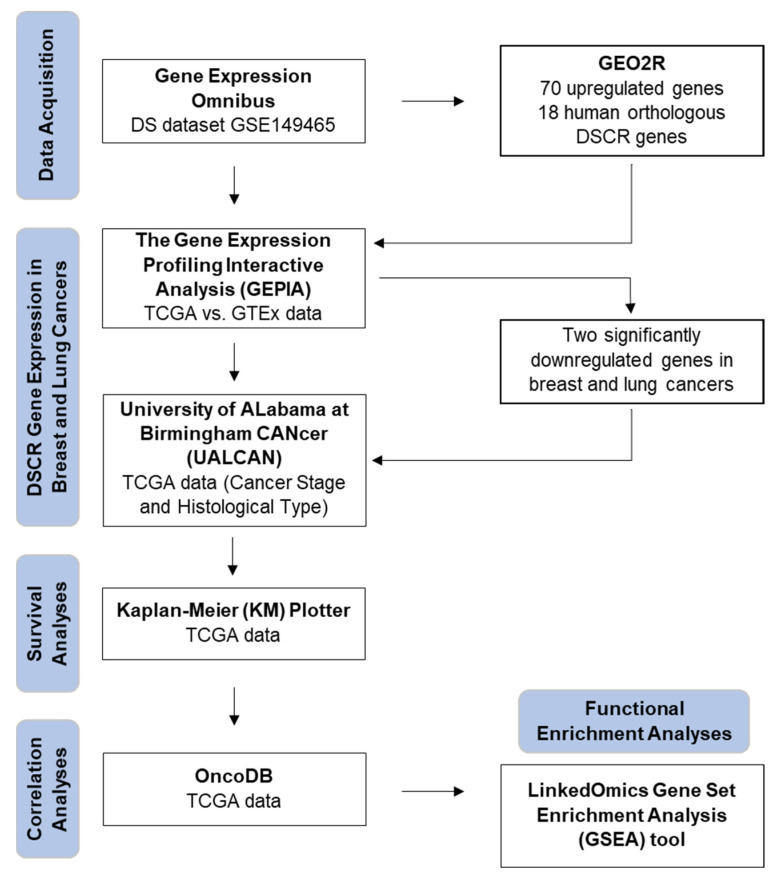
Flow diagram of bioinformatics approach used in study.

Although the inverse comorbidity between DS and solid tumors has been established, only a few studies have been conducted to understand the molecular processes and precise biological mechanism(s) responsible for this observation [18,19,20,21]. Using a systematic computational approach (Figure 1), we identified genes within the DSCR which may be protective against breast and lung cancers.

## 2. Materials and Methods

### 2.1. Gene Expression Data Acquisition

DS dataset GSE149465, submitted by Duchon et al. (2021) [22], was extracted from the Gene Expression Omnibus (GEO) database (http://www.ncbi.nlm.nih.gov/geo, accessed on 16 December 2022) for this study. The dataset contains gene expression data of the hippocampi of five male control littermates (Ts65Dn wildtype (WT)) and five male mutant trisomic mice (Ts65Dn mutant trisomy) analyzed with an Affymetrix GeneChip^®^ Mouse Gene 1.0 ST Array System (Santa Clara, CA, USA). 

In mice, there are three independent chromosomal regions homologous to HSA21. They are altogether made up of 158 protein-coding homologous genes out of the 228 protein-coding genes identified on HSA21. The largest region is found on *Mus musculus* chromosome 16 (Mmu16) with 119 orthologous genes. The other parts are found on mouse chromosome 17 (Mmu17) with 19 homologous genes, and mouse chromosome 10 (Mmu10) with 37 genes. 

The most widely used DS murine model is the Ts(1716)65Dn (noted Ts65Dn) mouse. It has a supplementary mini-chromosome containing the centromeric region of Mmu17 and the telomeric fragment of Mmu16. Of all the DS murine models (Ts1Cje, Ts65Dn and Dp(16)1/Yey), the Ts65Dn mouse is the only DS model that segregates an extra mouse chromosome; all other models are made by direct duplication of a mouse chromosome segment that is orthologous to HSA2 [23]. All the DS mouse models used in the study by Duchon et al. (2021) displayed defects in behavior and cognition upon behavioral phenotyping and magnetic resonance imaging [22].

### 2.2. Identification of Differentially Expressed DSCR Genes

GEO2R (https://www.ncbi.nlm.nih.gov/geo/geo2r/, accessed on 16 December 2022) was used to identify differentially expressed genes (DEGs) between the Ts65Dn mutant trisomy and Ts65Dn WT groups. Genes with adjusted *p* value < 0.05 and |logFC| ≥ 1 were considered DEGs. 

### 2.3. Analyses of DSCR Gene Expression in Breast and Lung Cancers

The Gene Expression Profiling Interactive Analysis (GEPIA2) webserver (http://gepia2.cancer-pku.cn/, accessed on 5 January 2023) [24] was used to evaluate the mRNA expression of the differentially expressed DSCR genes in breast and lung cancers. Gene expression data from The Cancer Genome Atlas (TCGA) (representing tumor tissues) and the Genotype-Tissue Expression (GTEx) project (representing normal tissues) were analyzed. Statistical significance was set at *p* value < 0.01 and |Log2FC| > 1.

### 2.4. Gene Expression Analyses Based on Cancer Stage and Subtype

The expression profiles of the genes of interest were further evaluated in human breast and lung cancers based on stage and histological type using TCGA data in the University of ALabama at Birmingham CANcer (UALCAN) data analysis portal (http://ualcan.path.uab.edu/, accessed on 5 January 2023) [25,26]. Statistical significance was achieved when *p* value was ≤0.05.

### 2.5. Survival Analyses

The prognostic values of the genes of interest in breast and lung cancer patients were examined using TCGA data in the Kaplan–Meier (KM) Plotter interactive web resource (https://kmplot.com/, accessed on 5 January 2023) [27]. KM Plotter is a web-based tool applied to evaluate the effect of 54,675 genes on survival using 10,461 cancer samples, including 5143 breast and 2437 lung samples. The hazard ratio (HR) was calculated with a 95% confidence interval. Statistical significance was achieved when log-rank *p* value was ≤0.05.

### 2.6. Correlation Analyses

Pair-wise gene expression correlation analyses of the genes of interest were performed using TCGA data in the OncoDB webserver (http://oncodb.org/index.html, accessed on 5 January 2023) [28]. OncoDB combines RNA-seq, DNA methylation, and associated clinical data from over 10,000 cancer patients in the TCGA and GTEx studies to examine gene expression patterns that are connected to clinical cancer characteristics. Statistical significance was achieved when *p* value was ≤0.05.

### 2.7. Functional Enrichment Analyses

The Gene Set Enrichment Analysis (GSEA) tool in the Link Interpreter module of the LinkedOmics webserver (http://www.linkedomics.org/admin.php, accessed on 7 January 2023) [29] was used to perform functional enrichment analyses of the genes of interest and their co-expressed genes. The LinkedOmics database is a web-based platform for analyzing 32 TCGA cancer-associated multi-dimensional datasets. To clarify the biological functions and signaling pathways that are regulated by the genes of interest and their co-expressed genes, we annotated each gene based on Gene Ontology (GO), Kyoto Encyclopedia of Genes and Genomes (KEGG) pathway and Panther pathway. For GO functional analyses, the gene functions were classified into biological process, cellular component, and molecular function.

## 3. Results

### 3.1. DEGs between Ts65Dn Mutant and Ts65Dn WT Groups

**Figure 2:**
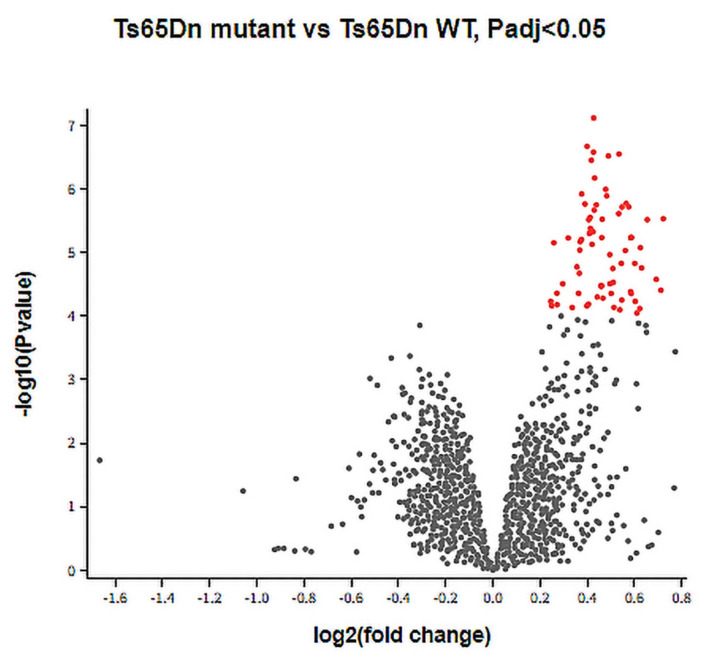
DEGs between Ts65Dn mutant and Ts65Dn WT groups. A volcano plot showing DEGs between the Ts65Dn mutant and Ts65Dn WT groups in the GSE149465 dataset. Grey dots: genes with no significant difference between groups, red dots: significantly upregulated DEGs based on adjusted *p* value < 0.05 and |logFC| ≥ 1.

Analysis of the GSE149465 dataset revealed a total of 70 genes that are differentially expressed between the Ts65Dn mutant and Ts65Dn WT groups. All the DEGs were significantly upregulated genes, with no significantly downregulated genes (Figure 2). Of the 70 significantly upregulated genes, 18 were identified to be human orthologous DSCR genes and were used for further analyses (Table 1).

### 3.2. Differential Expression of DSCR Genes in Breast and Lung Cancers

**Figure 3:**
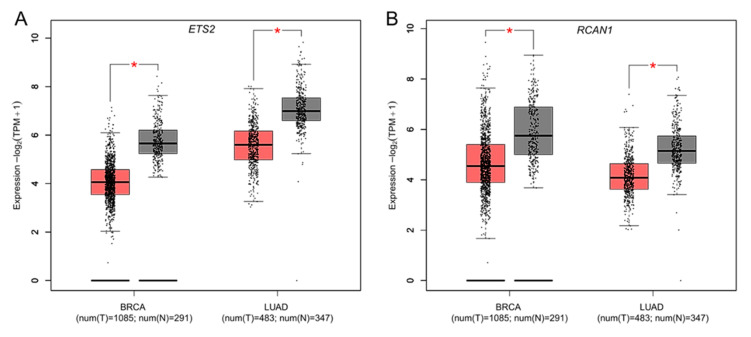
Differential expression of DSCR genes in breast and lung cancers. Boxplots represent mRNA expression of (**A**) *ETS2* and (**B**) *RCAN1* in breast and lung cancers. Red box: tumor samples, black box: normal samples, BRCA: breast invasive carcinoma, LUAD: lung adenocarcinoma, T: tumor, N: normal, * *p* value < 0.01, |Log2FC| > 1.

**Figure 4:**
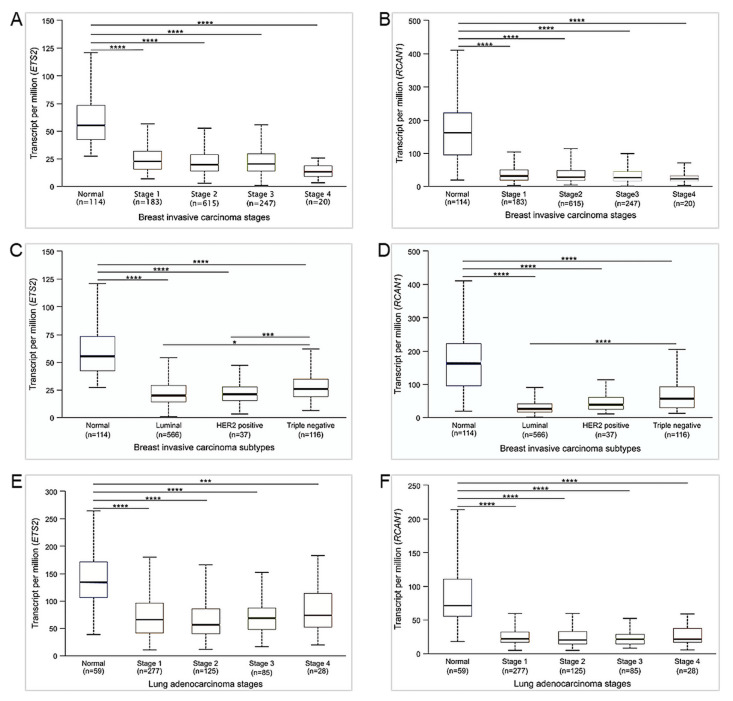
Expression of *ETS2* and *RCAN1* in breast and lung cancers based on stages and subtypes. Boxplots represent mRNA expression of (**A**) *ETS2* and (**B**) *RCAN1* in breast cancer stages; (**C**) *ETS2* and (**D**) *RCAN1* in breast cancer subtypes; and (**E**) *ETS2* and (**F**) *RCAN1* in lung cancer stages. * *p* ≤ 0.05, *** *p* ≤ 0.001, **** *p* ≤ 0.0001. Non-significant differences are not shown.

The mRNA expression levels of the 18 DSCR genes in human breast and lung cancers from TCGA and GTEx projects were analyzed using GEPIA2. Of these genes, *ETS2* and *RCAN1* were determined to be significantly downregulated in both cancers (Figure 3), suggesting that they may act as tumor suppressors against breast and lung cancers. All other genes showed no significant difference in their expression between tumor and normal samples (Supplementary Figure S1A–R). The UALCAN analyses showed that *ETS2* and *RCAN1* expressions were not influenced by breast cancer stages (Figure 4A,B). However, their expression levels were higher in triple-negative breast cancer (TNBC) compared to luminal and HER2-positive breast cancers (Figure 4C,D). Similarly, *ETS2* and *RCAN1* expressions were not influenced by lung cancer stages (Figure 4E,F).

### 3.3. ETS2 and RCAN1 Are Associated with Low Overall Survival in Breast and Lung Cancers

**Figure 5:**
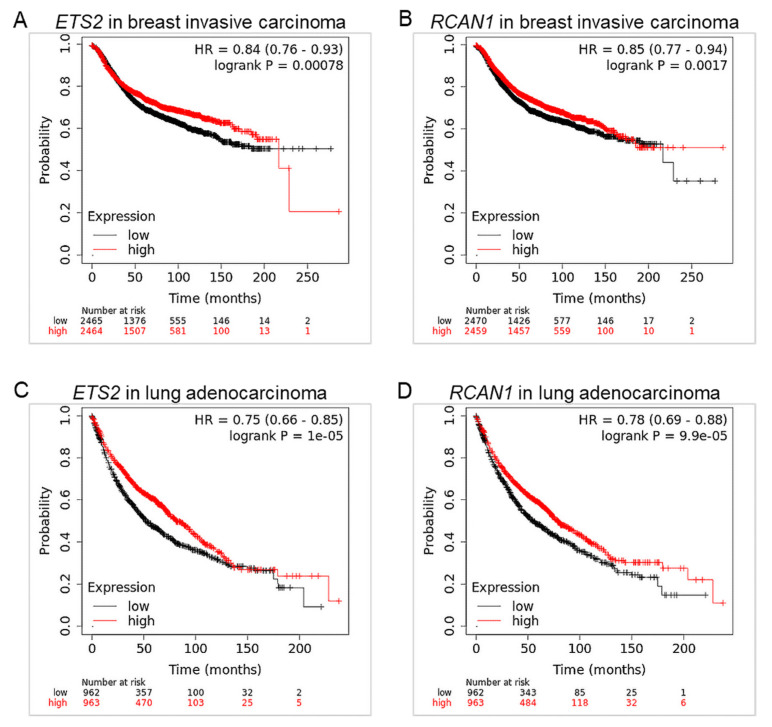
*ETS2* and *RCAN1* are associated with low overall survival in breast and lung cancers. Survival curves of (**A**) *ETS2* and (**B**) *RCAN1* in breast cancer; and (**C**) *ETS2* and (**D**) *RCAN1* in lung cancer. Red curve: high expression, black curve: low expression, HR: hazard ratio, long rank *p* value ≤ 0.05.

The associations between *ETS2* and *RCAN1* and patient survival in breast and lung cancers were investigated using KM Plotter. The survival curves show that in breast cancer, low expression of *ETS2* and *RCAN1* is associated with a significantly lower probability of survival (Figure 5A,B). Similar results were observed in the lung cancer cohort (Figure 5C,D), suggesting that *ETS2* and *RCAN1* expression affect survival outcomes in breast and lung cancers.

### 3.4. ETS2 and RCAN1 Expression Are Positively Correlated in Breast and Lung Cancers

**Figure 6:**
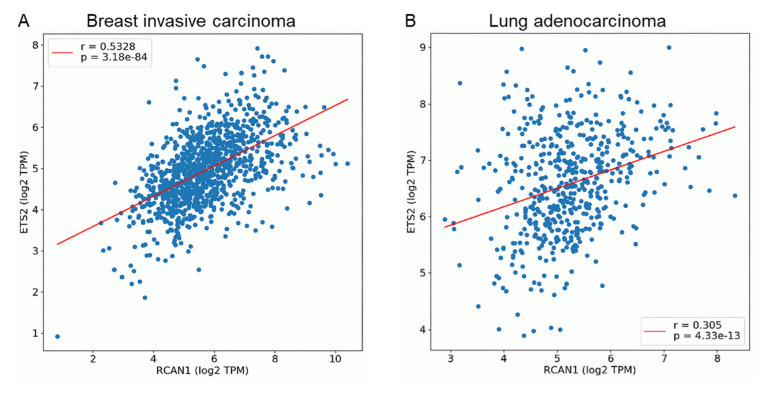
*ETS2* and *RCAN1* expression are positively correlated in breast and lung cancers. Scatter plots shows the correlation of gene expression of *ETS2* and *RCAN1* in (**A**) breast and (**B**) lung cancers. Blue dots represent individual mRNA levels expressed as log2 transcript count per million. r: correlation coefficient, *p* value ≤ 0.05.

We further explored the possibility of co-expression of *ETS2* and *RCAN1* using OncoDB. Pair-wise gene expression correlation analyses revealed that *ETS2* and *RCAN1* are positively correlated in breast and lung cancers, with a stronger correlation seen in breast cancer (Figure 6).

### 3.5. Functional Analyses of ETS2 in Breast and Lung Cancers

**Figure 7:**
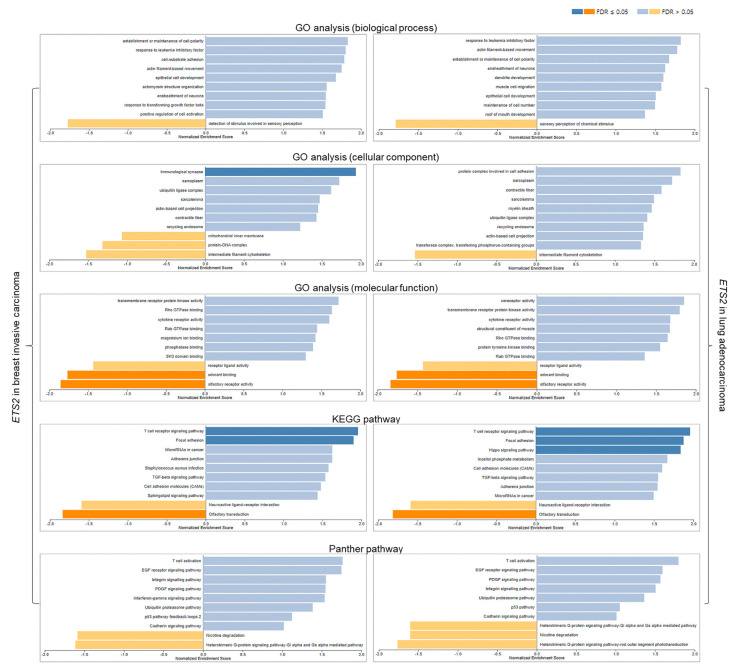
Functional analyses of *ETS2* in breast and lung cancers. Blue bars: gene clusters positively correlated with *ETS2*, orange bars: gene clusters negatively correlated with *ETS2*. FDR: false discovery rate.

The GO analyses for cellular components and molecular function showed that *ETS2* is involved in the positive regulation of immunological synapses and the negative regulation of odorant binding and olfactory receptor activity in breast and lung cancers. KEGG pathway analysis also showed that in both cancers, *ETS2* may positively regulate T-cell receptor signaling, focal adhesion, Hippo signaling pathway, and transforming growth factor β (TGF-β) signaling, and may negatively influence olfactory regulation and neuroactive ligand–receptor interaction. Furthermore, Panther pathway analysis revealed that in breast and lung cancers, *ETS2* may positively influence T-cell activation as well as the epidermal growth factor (EGF) receptor, integrin, platelet-derived growth factor (PDGF), interferon-γ (IFN-γ), p53, and cadherin signaling pathways. Nicotine degradation and the heterotrimeric G-protein signaling pathway may also be negatively regulated by *ETS2* in both cancers (Figure 7).

### 3.6. Functional Analyses of RCAN1 in Breast and Lung Cancers

**Figure 8:**
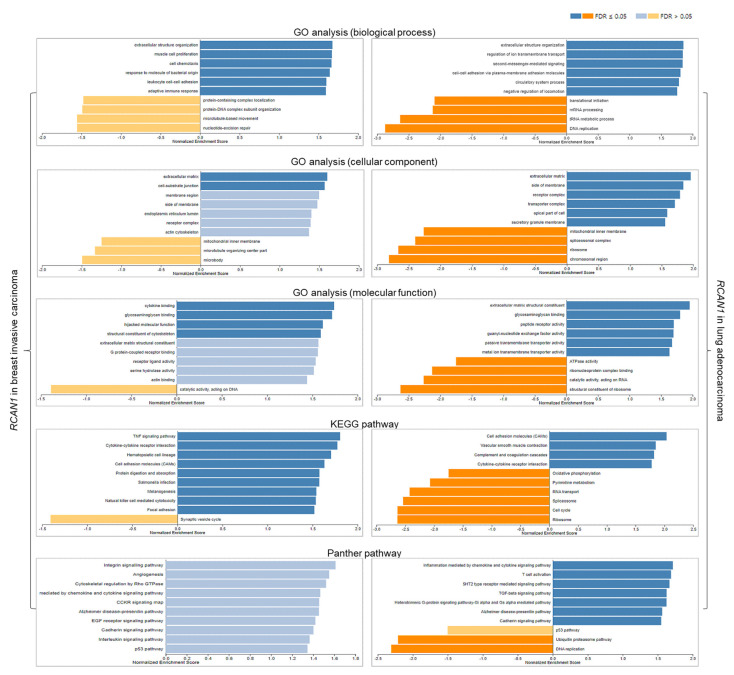
Functional analyses of *RCAN1* in breast and lung cancers. Blue bars: gene clusters positively correlated with *RCAN1*, orange bars: gene clusters negatively correlated with *RCAN1*. FDR: false discovery rate.

GO analyses for biological processes showed that in breast and lung cancers, *RCAN1* may positively regulate extracellular structure organization, myocyte proliferation, cell chemotaxis, cell–cell adhesion, and glycosaminoglycan binding while negatively regulating translational initiation, mRNA processing, tRNA metabolic process, and DNA replication. The cellular component analysis also showed that in both cancers, *RCAN1* is significantly enriched in the extracellular matrix, receptor and transporter complexes, and cell–substrate junction. GO for molecular function showed that in breast and lung cancers, *RCAN1* may be involved in cytokine and glycosaminoglycan binding. KEGG pathway analysis revealed that in both cancers, *RCAN1* may be involved positive regulation of cytokine–cytokine regulation, TGF-β signaling, tumor necrosis factor (TNF) signaling, and cell adhesion, and the negative regulation of pyrimidine metabolism, cell cycle, and DNA replication. Moreover, Panther pathway analysis revealed that in both cancers, *RCAN1* may be involved positive regulation of T-cell activation and angiogenesis as well as the integrin, EGF receptor, TGF-β, and cadherin signaling pathway while negatively regulating the ubiquitin–proteasome pathway and DNA replication. Additionally, *RCAN1* may positively influence the p53 pathway in breast cancer, but negatively influence the p53 pathway in lung cancer (Figure 8).

## 4. Discussion

Globally, DS is the most common genetic disorder, and research is still ongoing to fully understand the syndrome. An inverse comorbidity has been observed between DS and solid tumors such as breast and lung cancers, offering a new direction for understanding the molecular mechanisms of DS as well as these cancers. The HSA21, which is principal in DS, has been determined to harbor a DSCR composed of approximately 33 genes which may be responsible for the characteristic features of DS. The DSCR genes are also theorized to act as tumor suppressors against solid tumors [4]. Nonetheless, the protective roles of the DSCR genes against solid tumors have not been elucidated. Research on the intriguing inverse comorbidity between some cancers and DS may result in new cancer prevention and treatment strategies. In this in silico study, we identified two DSCR genes (*ETS2* and *RCAN1*) that are upregulated in DS mouse models but are significantly downregulated in human breast and lung cancer tissues compared to normal tissues and are associated with cancer patient survival.

The ETS (erythroblast transformation-specific) family of transcription factors are composed of a total of 28 ETS proteins in humans. They regulate numerous genes that are involved in cell proliferation, differentiation, development, angiogenesis, and apoptosis. ETS1 and ETS2 are representative members of the ETS family and are downstream effectors of the RAS/RAF/ERK pathway [30]. Depending on the biological event, the *ETS2* gene functions in tumor suppression or oncogenesis. On one hand, ETS2 suppresses tumors by recruiting co-repressors and activating gene transcription [31]. On the other hand, ETS2 may contribute to tumorigenesis by stabilizing gain-of-function mutant p53, inducing cyclin D1 promoter activity, and binding ETS sites on the hTERT promoter region [30]. In breast cancer cells, ETS2 may inhibit invasive properties [32]. Conversely, transfection of MCF-12A breast cancer cells with ETS2 promotes proliferation, adhesiveness, and invasiveness [33]. Furthermore, ETS2 interacts with c-Myc oncogene to maintain hTERT expression in breast cancer [34], sustaining the replicative potential of the cancer cells. From our analyses, *ETS2* levels were associated with patient survival but not cancer stage, suggesting that *ETS2* is associated with the development but not the progression of breast and lung cancers. Furthermore, *ETS2* levels were lower in luminal and HER2-positive breast cancers compared to TNBC, indicating that it may play a role in the molecular profiles that characterize these breast cancer subtypes. Further research is needed to ascertain the biological conditions that instigate the opposing roles of ETS2 in breast cancer. ETS1/2 proteins also regulate the expression of dual specificity phosphatase 6 protein which suppresses lung cancer progression by inhibiting oncogenic ERK signaling [35]. In human non-small cell lung cancer (NSCLC), Kabbout et al. (2013) demonstrated that ETS2 acts as a tumor suppressor by inhibiting MET proto-oncogene [36]. Thus, ETS2 may at least partially protect DS individuals from developing lung cancer.

*RCAN1* gene, also known as *DSCR1* (Down syndrome critical region 1) gene, exists as two isoforms in human tissues: *RCAN1.1* and *RCAN1.4*. Post-transcriptional modifications result in the generation of two versions of *RCAN1.1*, namely *RCAN1.1L* (the major isoform) and *RCAN1.1S* (the truncated form of *RCAN1.1 L*). RCAN1 is an endogenous inhibitor of calcineurin dephosphorylation, which is critical for the activation and nuclear translocation of nuclear factor of activated T cells (NFAT) [37]. Since NFAT promotes cancer cell proliferation and invasiveness, RCAN1 serves as a tumor suppressor [37,38,39]. RCAN1 expression in breast cancer tissues is lower than in adjacent healthy tissues [40], and its isoform RCAN1-4 represses breast cancer progression by inhibiting calcineurin/NFAT2 signaling [41]. From this study, *RCAN1* levels were associated with patient survival but not cancer stage, suggesting that *RCAN1* is associated with the development but not the progression of breast and lung cancers. Similar to *ETS2*, *RCAN1* expression was downregulated in luminal and HER2-positive breast cancers compared to TNBC, suggesting that it may influence the molecular features of breast cancer subtypes. RCAN1 reduces the aggressiveness of lung cancer cell lines by inhibiting the calcineurin/NFAT pathway [21,42]. Additionally, RCAN1 overexpression suppresses lung metastases by modulating the CN/NFAT/angiopoietin 2 signaling axis in lung endothelium [43]. Moreover, Kim et al. (2020) detected lower RCAN1-4 levels in NSCLC tissues compared to healthy lung tissues [44]. This suggests that RCAN1 acts as a tumor suppressor in lung cancer.

In this study, a positive correlation between *ETS2* and *RCAN1* was observed in breast and lung cancers. A previous study which assessed the frequencies of deleted regions in HSA21 in solid tumors reported that the *RCAN1* gene is deleted in Wilms tumors and downregulated (but not deleted) in breast and lung cancers [45]. Another study discovered that in breast and lung cancers, *ETS2* amplification (but not deletion) is the only type of genetic alteration [46]. As such, we hypothesize that other mechanisms rather than large gene deletions are responsible for the concurrent lowered expression of both genes in these cancers. Bassuk et al. (1997) demonstrated that a physical interaction occurs between multiple ETS and NF-kB/NFAT proteins in activated normal human T cells [47]. Tsao et al. (2013) also revealed that in primary Th1 cells, ETS1—an ETS2 homolog—physically and functionally interacts with NFAT to modulate IL-2-mediated immune responses [48]. Given the role of RCAN1 in the regulation of NFAT, it is likely that ETS2 and RCAN1 function cooperatively in cellular responses. Indeed, Luo et al. (2021) determined that ETS2 can bind to the *RCAN1* promoter and can form a complex with NFAT to transactivate *RCAN1* [49]. This suggests that ETS2 and RCAN1 are co-expressed and may possess complementary functions in the development of breast and lung cancers.

The functional enrichment analysis in this study showed that ETS2 may participate in T-cell receptor signaling, regulation of immunological synapses, focal adhesion, the Hippo signaling pathway, TGF-β signaling, EGF receptor signaling pathway, integrin signaling pathway, PDGF signaling pathway, and IFN-γ signaling pathway. These signaling pathways have been shown to affect the development and progression of breast and lung cancers [50,51], suggesting that ETS2 may contribute to the biology and immune landscape of breast and lung cancers. The functional enrichment analysis also revealed that RCAN1 may be involved in cytokine regulation, T-cell activation, TNF signaling, TGF-β signaling, integrin signaling pathway, angiogenesis, EGF receptor signaling pathway, cadherin signaling pathway, and the p53 pathway in breast and lung cancers. These biological events are critical for the pathogenesis and advancement of breast and lung cancers [50,51]. Thus, RCAN1 expression may be critical for biological processes and immune responses to influence breast and lung tumorigenesis.

In conclusion, *ETS2* and *RCAN1*, which are significantly upregulated in DS mouse models, are downregulated in human breast and lung cancers and are associated with patient survival. Considering their expression patterns in DS and breast and lung cancers, as well as their co-expression in these cancers, we hypothesize that *ETS2* and *RCAN1* are at least partly responsible for the low incidences of breast and lung cancers in DS. It is, however, necessary to note that the *RCAN1* gene assessed in this study could not be verified as the *RCAN1.1* or *RCAN1.4* isoform. Furthermore, the tissue-specific expression patterns of the genes in the hippocampi of the Ts65Dn models may not reflect patients’ breast and lung tumor expression data. Therefore, experimental research is necessary to test this hypothesis and to fully understand the roles of these genes in DS and breast and lung cancers.

## Figures and Tables

**Table 1:** Upregulated DSCR genes in the Ts65Dn mutant group compared to the Ts65Dn WT group.

Gene Symbol	Gene Name	Chromosomal Position	
		Human	Mouse
*Bace2*	β-site app-cleaving enzyme 2	chr16:97157942-97244136	chr21:39556492-39670904
*Brwd1*	Bromodomain and WD repeat-containing protein 1	chr16:95793292-95883628	chr21:37568748-37698102
*B3galt5*	β-1,3-galactosyltransferase 5	chr16:96037001-96121059	chr21:38042256-38057589
*Cbr1*	Carbonyl reductase 1	chr16:93404725-93407393	chr21:34453172-34455941
*Cbr3*	Carbonyl reductase 3	chr16:93480103-93487878	chr21:34518104-34529412
*Dopey2*	Dopey protein 2	chr16:93508792-93607476	
*Dscam*	Down syndrome cell adhesion molecule	chr16:96392040-96971952	chr21:38398213-39235595
*Dscr3*	Down syndrome critical region gene 3	chr16:94298642-94327689	chr21:37223425-37267532
*Dyrk1a*	Dual-specificity tyrosine-phosphorylation-regulated kinase 1a	chr16:94370869-94495926	chr21:35802642-35897561
*Ets2*	Ets proto-oncogene 2 transcription factor	chr16:95502942-95522095	chr21:37188977-37208711
*Hlcs*	Holocarboxylase synthetase	chr16:93931271-94091145	chr21:35130820-35348858
*Kcnj6*	Potassium inwardly-rectifying channel 6	chr16:94561928-94798555	chr21:36006636-36299912
*Morc3*	Microrchidia 3	chr16:93629009-93672961	chr21:34702785-34759216
*Pigp*	Phosphatidylinositol N-acetylglucosaminyltransferase subunit P	chr16:94165494-94172701	chr21:35447565-35455253
*Psmg1*	Proteasome assembly chaperone 1	chr16:95781133-95792160	chr21:37559338-37568021
*Rcan1*	Regulator of calcineurin 1	chr16:92188843-92196965	chr21:32899008-32908032
*Ttc3*	Tetratricopeptide protein ligase	chr16:94171477-94270079	chr21:35455544-35585333
*Wrb*	Tryptophan-rich basic protein	chr16:95946607-95959052	chr21:37764912-37900121

## Data Availability

The Down syndrome transcriptomics data referenced are available in public repository from the Gene Expression Omnibus (GEO) database.

## Supplementary Materials

The following supporting information can be downloaded at: https://www.mdpi.com/article/10.3390/genes14040800/s1, Figure S1: Differential expression of upregulated DSCR genes in breast and lung cancers. Table S1: Survival of breast cancer patients based on expression of DSCR genes. Table S2: Survival of lung cancer patients based on expression of DSCR genes.

## References

[1] CDC (2022). Facts about Down Syndrome|CDC.

[2] Bull M.J. (2020). Down Syndrome. N. Engl. J. Med..

[3] de Graaf G., Buckley F., Skotko B.G. (2021). Estimation of the Number of People with Down Syndrome in Europe. Eur. J. Hum. Genet..

[4] Antonarakis S.E., Skotko B.G., Rafii M.S., Strydom A., Pape S.E., Bianchi D.W., Sherman S.L., Reeves R.H. (2020). Down Syndrome. Nat. Rev. Dis. Prim..

[5] Vega Chromosome 21: 1-1—Chromosome Summary—*Homo sapiens*—Vega Genome Browser 68. http://vega.archive.ensembl.org/Homo_sapiens/Location/Chromosome?r=21.

[6] Osuna-Marco M.P., López-Barahona M., López-Ibor B., Tejera Á.M. (2021). Ten Reasons Why People with Down Syndrome Are Protected from the Development of Most Solid Tumors-A Review. Front. Genet..

[7] Di Cunto F., Berto G. (2013). Molecular Pathways of Down Syndrome Critical Region Genes. Down Syndrome.

[8] Allen E.G., Freeman S.B., Druschel C., Hobbs C.A., O’Leary L.A., Romitti P.A., Royle M.H., Torfs C.P., Sherman S.L. (2009). Maternal Age and Risk for Trisomy 21 Assessed by the Origin of Chromosome Nondisjunction: A Report from the Atlanta and National Down Syndrome Projects. Hum. Genet..

[9] Yoon P.W., Freeman S.B., Sherman S.L., Taft L.F., Gu Y., Pettay D., Flanders W.D., Khoury M.J., Hassold T.J. (1996). Advanced Maternal Age and the Risk of Down Syndrome Characterized by the Meiotic Stage of Chromosomal Error: A Population-Based Study. Am. J. Hum. Genet..

[10] Oliver T.R., Feingold E., Yu K., Cheung V., Tinker S., Yadav-Shah M., Masse N., Sherman S.L. (2008). New Insights into Human Nondisjunction of Chromosome 21 in Oocytes. PLoS Genet..

[11] Coppedè F. (2016). Segmental Trisomy of Murine Chromosome 16: A New Model System for Studying Down Syndrome. Arch. Toxicol..

[12] Shimada A. (2021). Profile of down Syndrome–Associated Malignancies: Epidemiology, Clinical Features and Therapeutic Aspects. Pediatr. Hematol. Oncol. J..

[13] Xavier A.C., Ge Y., Taub J.W. (2009). Down Syndrome and Malignancies: A Unique Clinical Relationship—A Paper from the 2008 William Beaumont Hospital Symposium on Molecular Pathology. J. Mol. Diagn..

[14] Hasle H., Friedman J.M., Olsen J.H., Rasmussen S.A. (2016). Low Risk of Solid Tumors in Persons with Down Syndrome. Genet. Med..

[15] Patja K., Pukkala E., Sund R., Iivanainen M., Kaski M. (2006). Cancer Incidence of Persons with Down Syndrome in Finland: A Population-Based Study. Int. J. Cancer.

[16] Yu Y.E., Xing Z., Do C., Pao A., Lee E.J., Krinsky-McHale S., Silverman W., Schupf N., Tycko B. (2020). Genetic and Epigenetic Pathways in Down Syndrome: Insights to the Brain and Immune System from Humans and Mouse Models. Prog. Brain Res..

[17] Dekker A.D., De Deyn P.P., Rots M.G. (2014). Epigenetics: The Neglected Key to Minimize Learning and Memory Deficits in Down Syndrome. Neurosci. Biobehav. Rev..

[18] Sussan T.E., Yang A., Li F., Ostrowski M.C., Reeves R.H. (2008). Trisomy Represses ApcMin-Mediated Tumours in Mouse Models of Down’s Syndrome. Nature.

[19] Yang A., Reeves R.H. (2011). Increased Survival Following Tumorigenesis in Ts65Dn Mice That Model Down Syndrome. Cancer Res..

[20] Reynolds L.E., Watson A.R., Baker M., Jones T.A., D’Amico G., Robinson S.D., Joffre C., Garrido-Urbani S., Rodriguez-Manzaneque J.C., Martino-Echarri E. (2010). Tumour Angiogenesis Is Reduced in the Tc1 Mouse Model of Down’s Syndrome. Nature.

[21] Shin J., Lee J.C., Baek K.-H. (2014). A Single Extra Copy of Dscr1 Improves Survival of Mice Developing Spontaneous Lung Tumors through Suppression of Tumor Angiogenesis. Cancer Lett..

[22] Duchon A., Del Mar Muniz Moreno M., Martin Lorenzo S., Silva de Souza M.P., Chevalier C., Nalesso V., Meziane H., Loureiro de Sousa P., Noblet V., Armspach J.-P. (2021). Multi-Influential Genetic Interactions Alter Behaviour and Cognition through Six Main Biological Cascades in Down Syndrome Mouse Models. Hum. Mol. Genet..

[23] Aziz N.M., Guedj F., Pennings J.L.A., Olmos-Serrano J.L., Siegel A., Haydar T.F., Bianchi D.W. (2018). Lifespan Analysis of Brain Development, Gene Expression and Behavioral Phenotypes in the Ts1Cje, Ts65Dn and Dp(16)1/Yey Mouse Models of Down Syndrome. Dis. Model. Mech..

[24] Tang Z., Kang B., Li C., Chen T., Zhang Z. (2019). GEPIA2: An Enhanced Web Server for Large-Scale Expression Profiling and Interactive Analysis. Nucleic Acids Res..

[25] Chandrashekar D.S., Karthikeyan S.K., Korla P.K., Patel H., Shovon A.R., Athar M., Netto G.J., Qin Z.S., Kumar S., Manne U. (2022). UALCAN: An Update to the Integrated Cancer Data Analysis Platform. Neoplasia.

[26] Chandrashekar D.S., Bashel B., Balasubramanya S.A.H., Creighton C.J., Ponce-Rodriguez I., Chakravarthi B.V.S.K., Varambally S. (2017). UALCAN: A Portal for Facilitating Tumor Subgroup Gene Expression and Survival Analyses. Neoplasia.

[27] Lánczky A., Győrffy B. (2021). Web-Based Survival Analysis Tool Tailored for Medical Research (KMplot): Development and Implementation. J. Med. Internet Res..

[28] Tang G., Cho M., Wang X. (2022). OncoDB: An Interactive Online Database for Analysis of Gene Expression and Viral Infection in Cancer. Nucleic Acids Res..

[29] Vasaikar S.V., Straub P., Wang J., Zhang B. (2018). LinkedOmics: Analyzing Multi-Omics Data within and across 32 Cancer Types. Nucleic Acids Res..

[30] Fry E.A., Inoue K. (2018). Aberrant Expression of ETS1 and ETS2 Proteins in Cancer. Cancer Rep. Rev..

[31] Ohtani N., Zebedee Z., Huot T.J., Stinson J.A., Sugimoto M., Ohashi Y., Sharrocks A.D., Peters G., Hara E. (2001). Opposing Effects of Ets and Id Proteins on p16^INK4a^ Expression during Cellular Senescence. Nature.

[32] Baker K.M., Wei G., Schaffner A.E., Ostrowski M.C. (2003). Ets-2 and Components of Mammalian SWI/SNF Form a Repressor Complex That Negatively Regulates the *BRCA1* Promoter. J. Biol. Chem..

[33] Schedin P.J., Eckel-Mahan K.L., McDaniel S.M., Prescott J.D., Brodsky K.S., Tentler J.J., Gutierrez-Hartmann A. (2004). ESX Induces Transformation and Functional Epithelial to Mesenchymal Transition in MCF-12A Mammary Epithelial Cells. Oncogene.

[34] Xu D., Dwyer J., Li H., Duan W., Liu J.-P. (2008). Ets2 Maintains HTERT Gene Expression and Breast Cancer Cell Proliferation by Interacting with C-Myc. J. Biol. Chem..

[35] Zhang Z., Kobayashi S., Borczuk A.C., Leidner R.S., Laframboise T., Levine A.D., Halmos B. (2010). Dual Specificity Phosphatase 6 (DUSP6) Is an ETS-Regulated Negative Feedback Mediator of Oncogenic ERK Signaling in Lung Cancer Cells. Carcinogenesis.

[36] Kabbout M., Garcia M.M., Fujimoto J., Liu D.D., Woods D., Chow C.-W., Mendoza G., Momin A.A., James B.P., Solis L. (2013). ETS2 Mediated Tumor Suppressive Function and MET Oncogene Inhibition in Human Non-Small Cell Lung Cancer. Clin. Cancer Res..

[37] Lao M., Zhang X., Yang H., Bai X., Liang T. (2022). RCAN1-Mediated Calcineurin Inhibition as a Target for Cancer Therapy. Mol. Med..

[38] Wang C., Saji M., Justiniano S.E., Yusof A.M., Zhang X., Yu L., Fernández S., Wakely P., La Perle K., Nakanishi H. (2017). RCAN1-4 Is a Thyroid Cancer Growth and Metastasis Suppressor. JCI Insight.

[39] Jin H., Wang C., Jin G., Ruan H., Gu D., Wei L., Wang H., Wang N., Arunachalam E., Zhang Y. (2017). Regulator of Calcineurin 1 Gene Isoform 4, Down-Regulated in Hepatocellular Carcinoma, Prevents Proliferation, Migration, and Invasive Activity of Cancer Cells and Metastasis of Orthotopic Tumors by Inhibiting Nuclear Translocation of NFAT1. Gastroenterology.

[40] Behtaji S., Ghafouri-Fard S., Sayad A., Sattari A., Rederstorff M., Taheri M. (2021). Identification of Oxytocin-Related LncRNAs and Assessment of Their Expression in Breast Cancer. Sci. Rep..

[41] Deng R., Huang J.-H., Wang Y., Zhou L.-H., Wang Z.-F., Hu B.-X., Chen Y.-H., Yang D., Mai J., Li Z.-L. (2020). Disruption of Super-Enhancer-Driven Tumor Suppressor Gene RCAN1. 4 Expression Promotes the Malignancy of Breast Carcinoma. Mol. Cancer.

[42] Ma N., Shen W., Pang H., Zhang N., Shi H., Wang J., Zhang H. (2017). The Effect of RCAN1 on the Biological Behaviors of Small Cell Lung Cancer. Tumor Biol..

[43] Minami T., Jiang S., Schadler K., Suehiro J.-I., Osawa T., Oike Y., Miura M., Naito M., Kodama T., Ryeom S. (2013). The Calcineurin-NFAT-Angiopoietin-2 Signaling Axis in Lung Endothelium Is Critical for the Establishment of Lung Metastases. Cell Rep..

[44] Kim D.H., Park S., Kim H., Choi Y.J., Kim S.Y., Sung K.J., Sung Y.H., Choi C.-M., Yun M., Yi Y.-S. (2020). Tumor-Derived Exosomal MiR-619-5p Promotes Tumor Angiogenesis and Metastasis through the Inhibition of RCAN1.4. Cancer Lett..

[45] Forés-Martos J., Cervera-Vidal R., Chirivella E., Ramos-Jarero A., Climent J. (2015). A Genomic Approach to Study Down Syndrome and Cancer Inverse Comorbidity: Untangling the Chromosome 21. Front. Physiol..

[46] Ren Y., Chen B., Zhang M. (2023). Distinct Prognostic and Immunological Roles of ETS1 and ETS2: A Pan-Cancer Analysis. Biomed Res. Int..

[47] Bassuk A.G., Anandappa R.T., Leiden J.M. (1997). Physical Interactions between Ets and NF-KappaB/NFAT Proteins Play an Important Role in Their Cooperative Activation of the Human Immunodeficiency Virus Enhancer in T Cells. J. Virol..

[48] Tsao H.-W., Tai T.-S., Tseng W., Chang H.-H., Grenningloh R., Miaw S.-C., Ho I.-C. (2013). Ets-1 Facilitates Nuclear Entry of NFAT Proteins and Their Recruitment to the IL-2 Promoter. Proc. Natl. Acad. Sci. USA.

[49] Luo Y., Jiang N., May H.I., Luo X., Ferdous A., Schiattarella G.G., Chen G., Li Q., Li C., Rothermel B.A. (2021). Cooperative Binding of ETS2 and NFAT Links Erk1/2 and Calcineurin Signaling in the Pathogenesis of Cardiac Hypertrophy. Circulation.

[50] Yuan M., Zhao Y., Arkenau H.-T., Lao T., Chu L., Xu Q. (2022). Signal Pathways and Precision Therapy of Small-Cell Lung Cancer. Signal Transduct. Target. Ther..

[51] Yousefnia S., Seyed Forootan F., Seyed Forootan S., Nasr Esfahani M.H., Gure A.O., Ghaedi K. (2020). Mechanistic Pathways of Malignancy in Breast Cancer Stem Cells. Front. Oncol..

