# Supplementary material for: Inverse Comorbidity between Down Syndrome and Solid Tumors: Insights from In Silico Analyses of Down Syndrome Critical Region Genes
Source: Genes (Basel). 2023 Mar 26;14(4):800. doi: 10.3390/genes14040800 (PMC10137705; doi:10.3390/genes14040800)
Supplement: Supplementary file 1 [file genes-14-00800-s001.zip › genes-2260488-supplementary.pdf]

SUPPLEMENTARY DATA

Fosu *et al.* Inverse comorbidity between Down syndrome and solid tumors: Insights from *in silico* analyses of Down syndrome critical region genes.

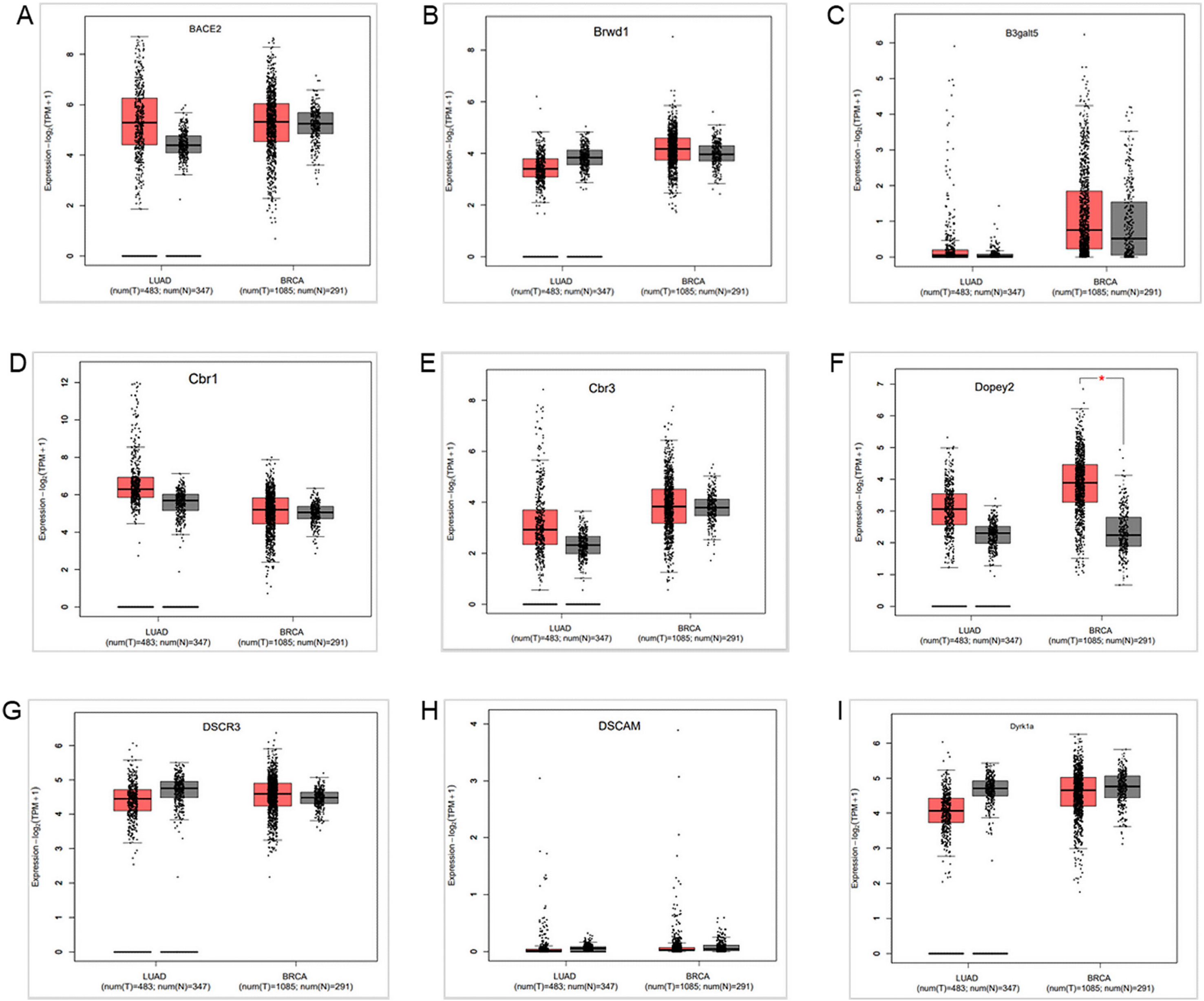

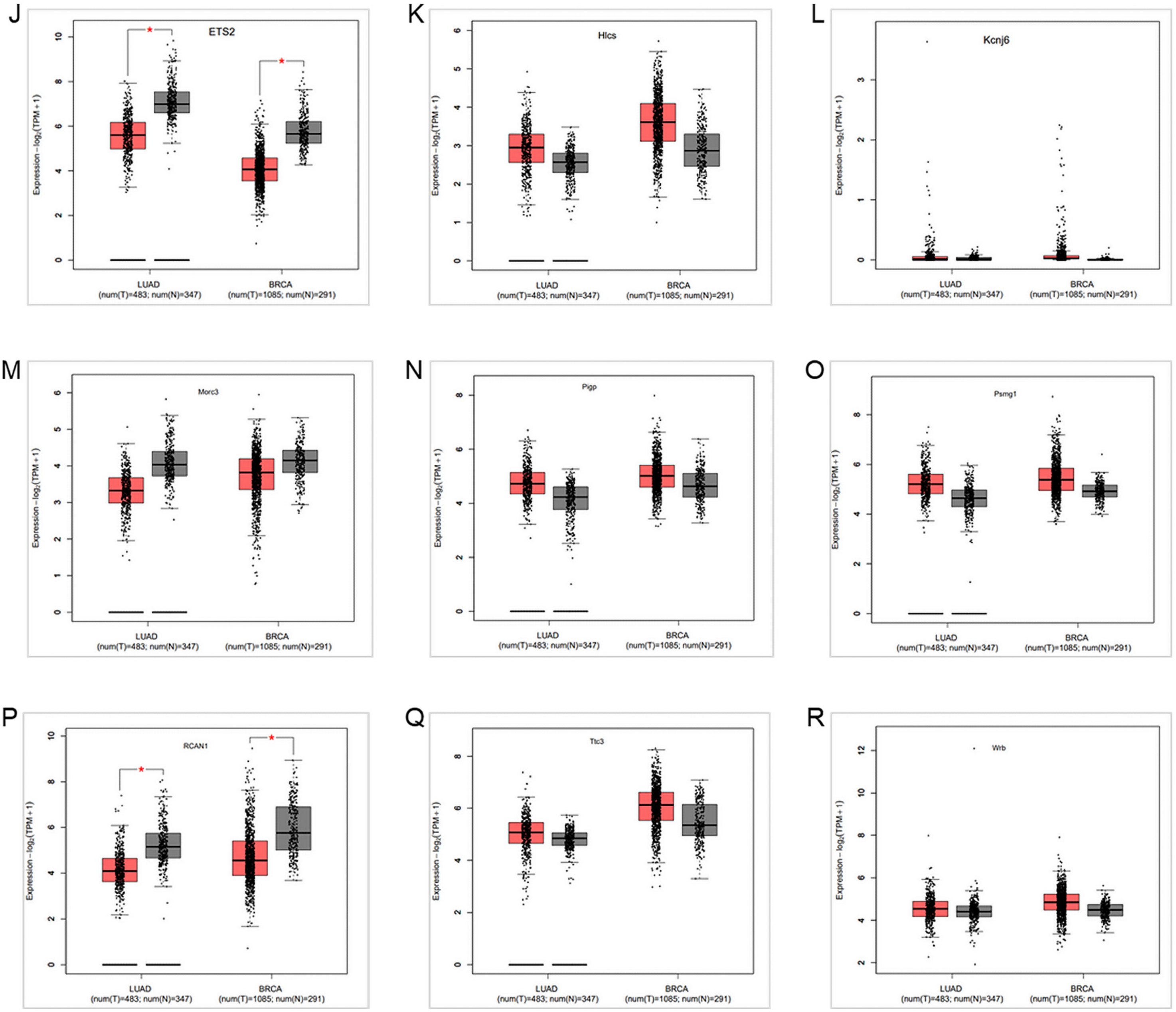

Supplemental Figure S1. Differential expression of upregulated DSCR genes in breast and lung cancers. Boxplots represent mRNA expression of (**A**) Bace2, (**B**) Brwd1, (**C**) B3galt5, (**D**) Cbr1, (**E**) Cbr3, (**F**) Dopey2, (**G**) Dscam, (**H**) Dscr3, (**I**) Dyrk1a, (**J**) Ets2, (**K**) Hlcs, (**L**) Kcnj6, (**M**) Morc3, (**N**) Pigp, (**O**) Psmg1, (**P**) Rcan1, (**Q**) Ttc3 and (**R**) Wrb in breast and lung cancers. Red box: tumor samples, black box: normal samples, BRCA: breast invasive carcinoma, LUAD: lung adenocarcinoma, T: tumor, N: normal, P value < 0.01, |Log2FC| < 1.

Supplemental Table S1. Survival of breast cancer patients based on expression of DSCR genes

Gene Symbol	Survival Mean		*P* value
	Low expression cohort (months)	High expression cohort (months)	
Bace2	148.53	122.3	0.0194
Brwd1	98.83	79.1	0.0633
B3galt5	129.1	131.37	0.0143
Cbr1	53.16	46	0.092
Cbr3	53.36	46	0.2473
Dopey2	228.85	191.21	0.6511
Dscam	36.9	40.3	0.1542
Dscr3	53.16	45.44	0.1238
Dyrk1a	131.97	122.3	0.274
Ets2	44.5	58.15	0.0008
Hlcs	215.2	115.37	0.0024
Kcnj6	142.23	115.4	0.1028
Morc3	248.5	116.4	0.0135
Pigp	219.77	115.37	0.0044
Psmg1	76	36.99	1E-15
Rcan1	43.44	58	0.0017
Ttc3	215.2	113.93	0.0415
Wrb	142.23	113.93	0.0028

Supplemental Table S2. Survival of lung cancer patients based on expression of DSCR genes

Gene Symbol	Survival Mean		*P* value
	Low expression cohort (months)	High expression cohort (months)	
Bace2	67	72.33	0.1963
Brwd1	47.37	50.03	0.1547
B3galt5	54.4	40.97	0.0165
Cbr1	53.33	45.23	0.2826
Cbr3	50.03	43.1	0.091
Dopey2	77.77	62.2	0.1069
Dscam	76	62.3	0.0354
Dcr3	80	62.3	0.0007
Dyrk1a	228.85	216.66	0.1444

Ets2	52	81	0.00001
Hlcs	54.07	42.93	0.3697
Kcnj6	50.93	38.23	0.0809
Morc3	42.27	57.5	0.0611
Pigp	50.03	47.37	0.2551
Psmg1	53.33	45.23	0.2217
Rcan1	54.3	79	0.000099
Ttc3	42.27	57.5	0.1386
Wrb	33.3	50.53	0.0225
